# CompMap: an allele-specific expression read counter based on competitive mapping

**DOI:** 10.17912/micropub.biology.001599

**Published:** 2025-06-02

**Authors:** Santiago Sanchez-Ramirez, Asher Cutter

**Affiliations:** 1 Department of Ecology and Evolutionary Biology, University of Toronto, Toronto, Ontario, Canada

## Abstract

Gene regulatory changes acting *cis* and *trans* to a gene can be inferred with allele-specific expression (ASE) transcriptomes from interspecies and inter-population hybrids and their parents. Problems of mapping bias and excessive information loss, however, can arise unintentionally from cumbersome analysis pipelines. We introduce CompMap, a self-contained method in Python that generates allele-specific expression counts from genotype-specific alignments. CompMap sorts and counts reads, not just SNPs, by comparing read-mapping statistics to parental alignments within homologous regions. Ambiguous alignments resolve proportionally to allele-specific counts or statistically using a binomial distribution. Simulations with CompMap show low error rates in assessing regulatory divergence.

**Figure 1. Accuracy in allele-specific expression (ASE) increases with protein-coding divergence using CompMap:**
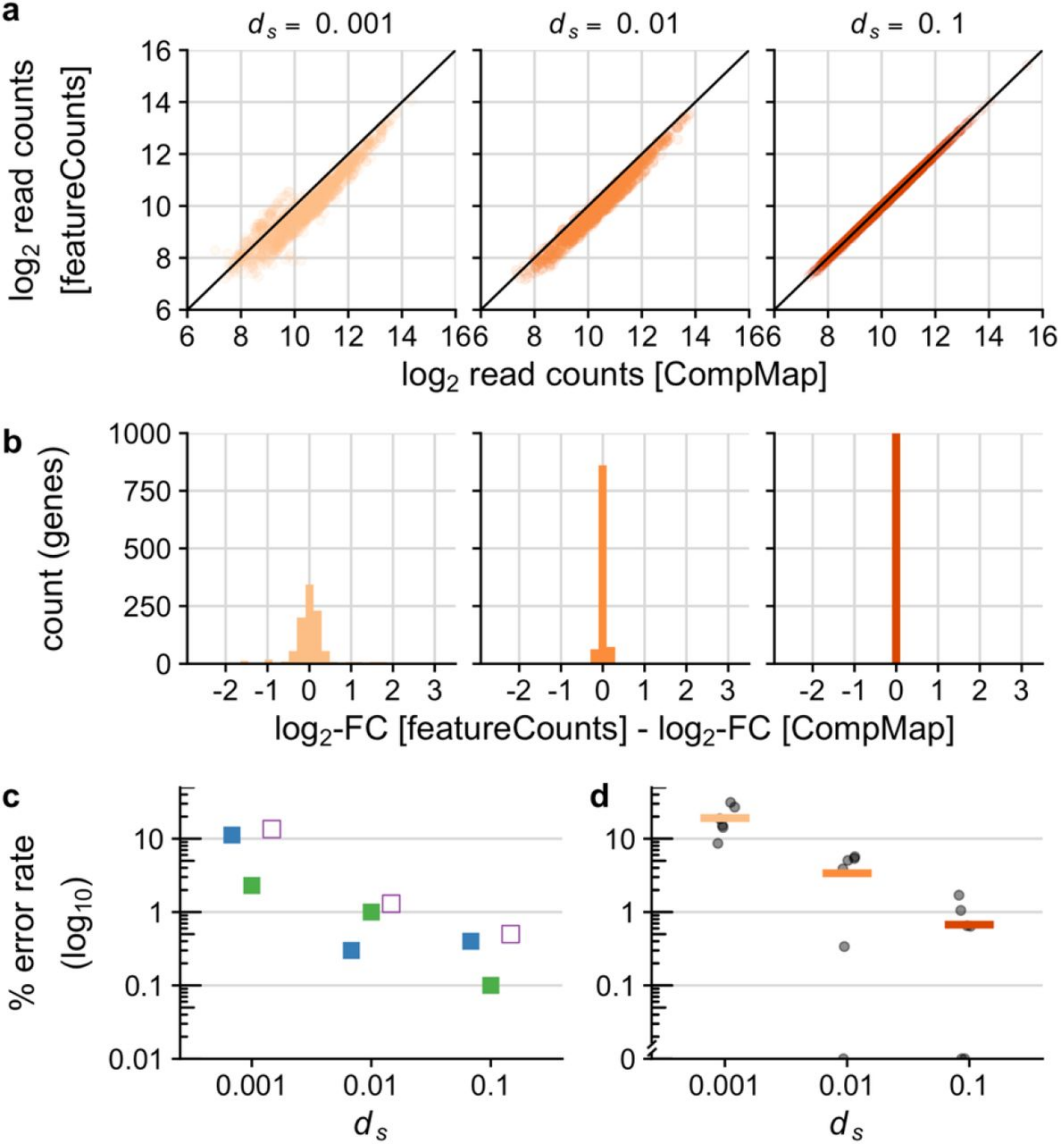
a) Biplot of log _2_ raw read counts between “true” allele-specific counts using featurecounts and “mixed” read counts using CompMap. b) Histograms of the difference in log _2_ -fold-change for ASE between featurecounts and CompMap (low neutral divergence *d _s_* =0.001: mean=0.0057, sd=0.508; moderate neutral divergence *d _s_* =0.01: mean=0.003, sd=0.068; high neutral divergence *d _s_* =0.1: mean=0.0017, sd=0.009). c) Estimates of the false negative error rate (blue), false positive error rate (green), and total error (open purple) to differential expression between alleles, and d) mean false negative rate across different regulatory divergence categories. See McManus et al. (2010) for a complete list of regulatory divergence categories. The “y” axis on c and d is based on the percent error plotted on a log _10_ scale.

## Description

Geneticists' long-standing quest to understand functional variation within genomes has led to the appreciation that changes in regulatory regions are crucial in fine-tuning developmental controls of gene expression to influence phenotypes, in addition to the effects of structural changes in protein-coding genes (The ENCODE Project Consortium, 2012; Signor and Nuzhdin, 2018). Non-coding mutations to cis-regulatory elements that are localized close to a gene and transregulatory factors that are encoded at a distant location in the genome provide the genetic variation for expression of a focal gene, which, in turn, can drive adaptive divergence between populations and between species (Jones et al., 2012; Wray, 2007). However, identifying genetic variants with cis-acting or trans-acting regulatory effects presents a serious challenge, as it is difficult to disentangle without explicit allele-specific expression (ASE) information (Signor and Nuzhdin, 2018).

Current ASE approaches include lengthy pipelines with multiple dependencies aimed at within-species datasets (Rozowsky et al., 2011), tissue-specificity (Pirinen et al., 2015), and cross-population samples (Fan et al., 2020), usually requiring reads to be mapped to a single genotype-specific reference, followed by variant-calling and phasing. That said, the field has been growing rapidly in the last decade, resulting in highly accurate tools (e.g., van de Gejin et al., 2015; Raghupathy et al., 2018; Adduri and Kim, 2024). Here, we present CompMap, a simple, self-contained, variant-calling-free Python tool for counting reads in ASE datasets. The motivation for developing CompMap stems from the need for a tool that circumvents complex bioinformatic pipelines while avoiding the limitations of variant-calling and generates read counts compatible with other downstream read-counting software.

## Methods

*Software*

The Python code with examples and installation instructions is available on the GitHub repository in "Extended Data". In brief, CompMap parses two F1 hybrid BAM files with RNA-seq reads mapped to each parental genome simultaneously and compares the alignment quality of each read against both references. Allele-specific reads are then "competitively" assigned, based on their relative mapping scores, and counted. CompMap is fully implemented in Python, relying on the pysam API library to read and parse BAM files and numpy (e.g., for binomial distribution corrections). It requires the following basic input files:

- BAM files with F1 hybrid (offspring) RNA-seq data mapped to each parental genotype
- BED files with genomic coordinates of the genes of interest, one per parental genotype

Preferably, BAM files are sorted and indexed. The 4th column of BED files should include the gene name and match for homologous regions between parental genomes (i.e., same name for both BED files). The -h or --help argument will print descriptions and other information to the screen. The two most important arguments are --AS_tag and --NM_tag, which indicate labels for the alignment score and number of mismatches tags, respectively. By default, CompMap will use tags from the STAR aligner (Dobin et al., 2012). The user can specify read-tag labels of different aligners, such as BWA (Li and Durbin, 2010) and Bowtie2 (Langmead and Salzberg, 2012). The --star argument will apply the NH tag when looking for multiple read matches, which may improve speed. Finally, users can specify the --binom tag to assign ambiguous read counts probabilistically to either one of the parental alleles. The binomial probability *p* describes the fraction of non-ambiguous reads with better matches to one parental genome for the corresponding gene (1- *p* better matching the other parental genome), and size defines the total number of ambiguously matching reads. The default behavior deterministically allocates reads with proportions p and 1- *p* without using binomial sampling.

We validated the read-counting performance of CompMap by comparing it to two commonly used differential expression tools: featureCounts (Liao et al., 2014) and HTseq-counts (Anders et al., 2015). Our results replicate the results of featureCounts and therefore recommend using this tool for read-counting parental RNA-seq data which can be used in combination with CompMap.

*Validation on simulated data*

Gene length and sequence differences between alleles can potentially impact the power to detect and quantify ASE with accuracy. To validate our approach, we simulated protein-coding sequence datasets of homologous alleles with synonymous site divergence. We neglected divergence at nonsynonymous sites because of their rarity in biological data of close relatives and to avoid assumptions about the strength and direction of selection. The presence of nonsynonymous differences in real data will make our power analysis conservative with respect to detecting and quantifying ASE.

The simulation procedure first drew 1000 random protein lengths from a Gamma distribution (scale=1000, shape=1.35; minimum length 300 aa). Non-stop codons were picked at random to form a "transcript", all of which started with the Methionine coding "ATG" and ended with any of the three stop codons. Synonymous substitutions were imposed to create a divergent version of every "transcript": each 4-fold, 3-fold, and 2-fold degenerate codon was allowed to mutate to a synonymous codon, selecting alternate codons with equal probability. Synonymous divergence used one of three rates: (1) high *d _s_* =0.1 substitutions/site (exemplifying interspecific variation; e.g., between species of *Caenorhabditis* ), (2) moderate *d _s_* =0.01 substitutions/site (exemplifying variation between divergent populations), and (3) low *d _s_* =0.001 substitutions/site (exemplifying intraspecific variation; e.g. humans). CompMap's GitHub repository contains the code for this simulation procedure in "Extended Data".

For each "allelic" transcript sequence, we then simulated RNA-seq reads using the R package polyester (Frazee et al., 2015). Fold-changes in expression were randomly sampled from an exponential distribution with sign (up- or down-regulation) assigned randomly. These generated data defined "true" ASE counts for comparison to estimates derived from CompMap. Similarly, we generated RNA-seq datasets representing expression differences in each homozygous parent. Fold-change values between "alleles" and "parents" were drawn from independent distributions.

To validate ASE counts from CompMap, we combined allele-specific reads into a single FASTA file for each replicate and mapped them to each transcript reference using BWA-MEM (Li and Durbin, 2010). We then generated BED files with coordinates for each transcript. The resulting BAM and BED files were fed to CompMap to perform ASE counts using the --NM_tag with NM, providing the BWA-specific read tag for number of mismatches.

Standard read counts for "parental" and true "allele" read data were performed with featureCounts (Liao et al., 2014). Raw read counts were then analyzed in R, and differential expression analyses were conducted with DESeq2 (Love et al., 2014). We followed McManus et al. (2010) to statistically assign genes to different regulatory divergence categories (e.g., *cis* -only, *trans* -only, *cis-trans* compensatory, *cis* x *trans* ).

*Statistical Analyses*

CompMap recovered high accuracy in allele-specific counts for all three simulated protein-coding datasets that spanned a 100-fold range of divergence (Figure 1a). Count accuracy was highest for genes with high divergence between alleles ( *d _s_* =0.1). In the more challenging case of low divergence ( *d _s_* =0.001), CompMap slightly overestimated the number of reads for a given allele (by a factor of approximately 1.75), being most pronounced among genes with a lower magnitude of expression, as expected. However, this effect does not strongly perturb analyses of differential expression (Figure 1b). High-divergence alleles also show the lowest variance in the ASE log _2_ -fold-difference between simulated allele-specific reads counted by featureCounts and CompMap (Figure 1b). Despite the low divergence dataset yielding the widest variability in estimated differential expression, the mean centered close to zero indicates little bias (Figure 1b).

Error rates to inferring differential expression between alleles declined approximately 10-fold with increasing divergence (Figure 1c). The power of CompMap to accurately classify genes into different regulatory divergence categories is greatest with high divergence between alleles (<2% false negative rates for the *d _s_* =0.1 dataset; <6% for intermediate *d _s_* =0.01) (Figure 1d). As expected, the highest inaccuracy occurred with low divergence ( *d _s_* =0.001 false negative rates ~20%).

Consequently, ASE reliability will be greatest for biological studies involving alleles with high sequence divergence, which will be challenging with neutral allele divergence ~0.1% (e.g., *d _s_* =0.001), as within humans (Perry et al., 2012) and *Caenorhabditis elegans* (Andersen et al., 2012). Moreover, although our specific use case involved high-quality reference genomes from *C. briggsae* and *C. nigoni* (Sánchez-Ramírez et al., 2021), we anticipate that differences in reference genome quality might also lead to mapping biases and other potential issues. Essential for such cases is a simulation framework for validation, as implemented in CompMap, to assess the power to quantify ASE.

CompMap's competitive read-mapping approach is sensitive and accurate, given sufficiently dense sequence differences between alleles. With CompMap, we 1) introduced a framework to reliably assess the power of recovering per-gene ASE read counts; 2) developed R code for simulation testing of coding sequence divergence; and 3) showed the best ASE inference with ≥1% allelic synonymous divergence. CompMap is ideal for RNA-seq datasets derived from interspecies hybrids (Sánchez-Ramírez et al., 2021), as well as within-species analysis of systems with high genetic diversity, including *Drosophila* and *Caenorhabditis* (Cutter et al., 2013).

## Data Availability

Description: GitHub repository. Resource Type: Software. DOI: https://doi.org/10.22002/07w0a-qtx12
